# Knowledge and Practice of Urinary Incontinence Management Among Nursing Professionals in Serbian Nursing Homes: A Multicentre Study

**DOI:** 10.3390/healthcare12232425

**Published:** 2024-12-03

**Authors:** Dragana Milutinović, Mira Novković Joldić, Dragana Simin, Dragana Živković

**Affiliations:** Department of Nursing, Faculty of Medicine, University of Novi Sad, 21000 Novi Sad, Serbia; mira.novkovic@mf.uns.ac.rs (M.N.J.); dragana.simin@mf.uns.ac.rs (D.S.)

**Keywords:** urinary incontinence, nursing homes, long-term care, nursing practice

## Abstract

Background/Objectives: Urinary incontinence (UI) is a prevalent condition among older adults, particularly in nursing home residents. Furthermore, it is associated with significant physical, emotional, and financial burdens. Effective management of UI depends on the knowledge and practices of nursing professionals, who are responsible for fundamental care. However, their knowledge and practice gaps remain challenging. Therefore, the study aimed to assess the knowledge and practice of nursing professional regarding urinary incontinence, to explore whether there are differences in knowledge and practice concerning their sociodemographic characteristics and to determine if there is a correlation between knowledge and practice regarding UI. Methods: A multicentre descriptive, cross-sectional study design following STROBE guidelines was conducted. The sample comprised 171 participants, and as research instruments, the Urinary Incontinence Quiz (UIQ) and the Urinary Knowledge and Practice Instrument (UKPI) were used. Results: The overall knowledge of nursing professionals about UI in older people was suboptimal, but most were familiar with the causes of UI, and that toilet training can improve incontinence. Nursing professionals with continuing education or interest in learning more about UI demonstrated significantly higher knowledge levels. Practice scores revealed that continence care interventions were often implemented, but documentation and support practices such as comprehensive toileting plans were less frequently performed. A weak but significant positive correlation was found between knowledge and practice. Conclusions: The study highlights substantial knowledge and practice gaps in UI management among nursing professionals in Serbia. The findings underscore the need for targeted educational interventions to improve UI care.

## 1. Introduction

Urinary incontinence (UI) has a high prevalence in the older population, especially among nursing home residents [1]. The prevalence of UI among nursing home residents ranges from 13 to 77%, depending on their age, functional physical and cognitive status, comorbidities, and the research methodology [2,3]. UI is a symptom considered to be a part of geriatric syndrome since many of the risk factors are not directly related to the genitourinary tract. Namely, its multifactorial aetiology includes physiological changes during ageing, polypharmacy, comorbidities, and functional and cognitive limitations [4]. In a multicentre study, Jerez-Roig et al. [1] carried out in Spanish nursing homes identified cognitive limitation, medications with anticholinergic activity and risk for sarcopenia as significant predictors of UI.

In general, the causes of UI can be classified into two categories: transient or reversible and chronic or established [3,5,6]. Two mnemonics (DIAPPERS and DISAPPEAR) have been created to make recalling causes of transient UI faster and easier. Ref. [6], whereas established UI often has structural causes and a neurological, urological, or gynaecological basis [3].

There are five types of established UI: stress, urgent, combined, overflow and functional [5,6]. According to the International Continence Society, stress urinary incontinence (SUI) is involuntary urine leakage due to coughing, sneezing or physical exertion [7,8]. Urge urinary incontinence (UUI) is the involuntary urine leakage accompanied by a strong urge and need to urinate. At the same time, mixed urinary incontinence (MUI) is the presence of involuntary urine leakage associated with the urgency to urinate, as well as straining, sneezing, or coughing [7,9]. UUI is part of a complex set of symptoms known as overactive bladder syndrome (OAB). Overactive bladder syndrome (OAB) is characterised by urinary urgency, with or without urgency urinary incontinence, usually with increased daytime frequency and nocturia, if there is no proven infection or other obvious pathology [10]. OAB, associated with UUI, is the most common comorbidity in nursing home users [4]. Overflow UI is the involuntary leakage of urine from an overdistended bladder due to impaired detrusor contractility and/or bladder outlet obstruction. In contrast, functional UI refers to non-urological causes of involuntary urine leakage, such as physical or cognitive barriers to toileting [11].

UI is a costly problem for nursing homes. It is estimated that the annual costs can be over five billion dollars when the expenses for single-use absorbent pads and other continence products, as well as laundering and time managing UI of nursing home employees, are included [12]. In addition to financial consequences, UI in nursing home residents increases the risk of dermatological problems, such as moisture-associated skin damage, perineal dermatitis, and pressure ulcers, followed by urinary tract infections (UTI), which worsen incontinence and are the leading cause of sepsis among this population [11]. Incontinent nursing home residents have a higher risk for falls and consequent injuries that may require immobilisation and indirectly increase episodes of incontinence [13]. In addition, UI could affect emotional well-being and lead to a loss of self-confidence, anxiety, and depression [14]. The physical and emotional complications of UI can lead to significant morbidity in terms of impaired quality of life. Therefore, the prevention and treatment of UI and its complications have been recognised as a priority for improving the care quality in nursing homes and residents’ quality of life [12].

Treatment of UI depends on the type of incontinence and should be considered within the institutional guidelines, as well as the resident’s preferences and comorbidities. Before starting the treatment, an assessment is needed, which includes a medical history, physical exam, and urinalysis. First-line treatment includes conservative management, second-line is medical therapy, and third-line treatment includes advanced therapeutic procedures, but there is not enough data on their outcomes in nursing home residents [4]. Conservative management of UI in nursing home residents involves behavioural interventions such as prompted and timed voiding, increasing physical activity, functional mobility, pelvic floor muscle exercise, fluid intake regiment, and their combination. Conservative management of UI is the nursing professional’s competence and responsibility [15].

However, despite the overwhelming negative impact of UI, it is still frequently undiagnosed, untreated, and overshadowed by other geriatric syndromes due to numerous coexisting comorbidities. On the one hand, this is particularly contributed by the fact that nursing home residents avoid reporting their continence problems due to feelings of shame and embarrassment [1]. On the other hand, a complete continence program, especially conservative measures, involves significant engagement of nursing home employees, particularly nursing professionals [16]. Still, one of the crucial barriers to implementing optimal UI conservative management is their insufficient knowledge [17,18,19,20].

In Serbian nursing homes, the nursing profession comprises nurses and nursing assistants. Nurses are responsible for all steps of the nursing process, from assessment to evaluation of the quality of the continence program. In contrast, nursing assistants are mainly involved in residents’ physical care and activities of daily living (ADLs), including changing incontinence products and completing a voiding diary. However, as a developing country, Serbia does not have a curriculum for urotherapists, i.e., advanced nurse practitioners who would replace physicians in designing the care process and promoting therapeutic continence in nursing home residents [21]. Therefore, this study had several aims: (a) to assess nursing professionals (nurses and nursing assistants) knowledge and practice regarding UI, (b) to explore whether there are differences in knowledge and practice regarding UI to their sociodemographic characteristics, and (c) to determine the correlation between knowledge and practice regarding UI.

## 2. Materials and Methods

### 2.1. Study Design and Setting

A multicentre descriptive, analytical, comparative, and correlational cross-sectional study was conducted. Data was collected through a self-assessment survey. Participants included nurses and nursing assistants employed in three nursing homes in Vojvodina (a province in the north of Serbia). The study followed Strengthening the Reporting of Observational Studies in Epidemiology (STROBE) guidelines.

### 2.2. Sample and Data Collection

**Figure 1:**
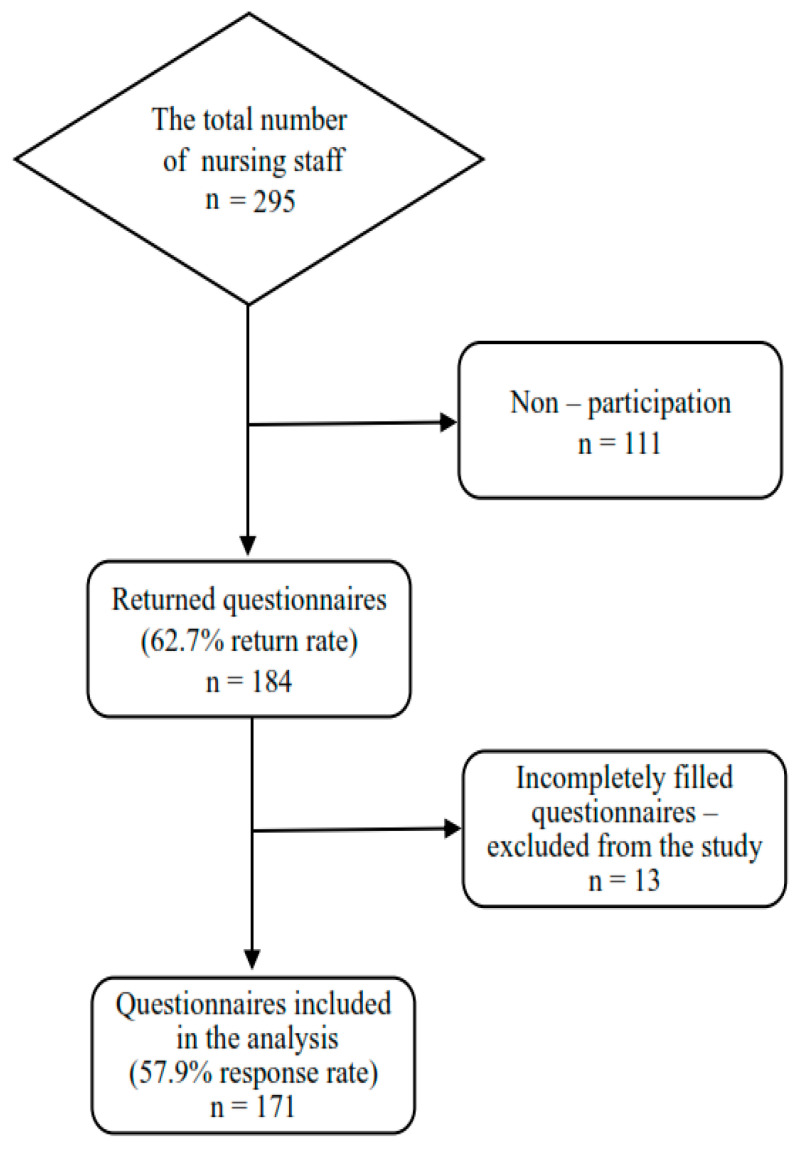
Flowchart of nursing professional inclusion.

Of the 295 questionnaires distributed, 184 were collected (62.7% return rate). Furthermore, 13 incompletely filled questionnaires were considered invalid and excluded from the study; therefore, 171 were included in the analysis (57.9% response rate) (Figure 1).

The convenience sample comprised n = 171 employees (n = 86 nurses, n = 85 nursing assistants). The criterion for inclusion in studies was that they had a minimum of six months of working experience. The study inclusion criteria were established since a minimum of six months of practice for the licensing exam is necessary.

The sample size was determined based on the number of employed nursing professionals who met the inclusion criteria (n = 295). Thus, using sample size software for cross-sectional studies, a sample of 168 is required for a 95% confidence interval with a 0.05 margin of error.

Data were collected using a paper version of the questionnaires during the day shift from February to May 2024. Cooperating with the head nurses of participating nursing homes, researchers (MNJ and DM) distributed questionnaires. All nurses and nursing assistants who met the inclusion criteria were invited to participate in the study. They were informed about the study before filling out the questionnaire, and after filling it out, they were deposited in a closed box placed in every nursing home to facilitate collection and ensure privacy and confidentiality.

### 2.3. Instruments

A general questionnaire for obtaining sociodemographic data, the Urinary Incontinence Quiz (UIQ) [22] and the Urinary Knowledge and Practice Instrument (UKPI) were used as study instruments [23].

The general questionnaire includes eight items for obtaining the following data: gender, age (year), profession (nurse/nursing assistants), total working experience (year), working experience in a nursing home (year), education (primary school/secondary school/high school or faculty), whether they had a continuing education course on urinary incontinence (yes/no) and interest in education about UI (yes/no).

The UIQ was designed by Branch et al. [22] and is the oldest questionnaire to assess UI knowledge. The questionnaire consists of 14 items (six correct and eight incorrect) grouped into four subscales: the relationship between ageing and UI (items 1,2); causes of UI (items 3, 8, 10, 12); the discussion between the patient and physician about UI (items 7, 9); and treatment and effects of UI (items 4–6, 11, 1 3, 14), each of which can be answered with agree, disagree and do not know. The score ranges from 0 to 14 or from 0% to 100% based on the total number of correct answers. A higher score indicates a higher level of knowledge and more positive attitudes towards UI. The measure of internal consistency for the entire scale in different studies ranged from α = 0.72 to 0.80 [24,25,26].

The UKPI was developed by Saxer et al. [23] and consists of a knowledge scale and a practice. The knowledge scale comprises 18 items related to older people’s urinary incontinence. The items are answered with true, false and do not know. True answers are scored with 1, and false and do not know with 0. A higher score indicates a higher level of knowledge about UI. The practice scale consists of 30 items grouped into four subscales (fluid intake and excretion—ten items, assessment and information—six items, documentation—six items and support—eight items) with 4-point Likert response categories (never done—0, sometimes done—1, often done—2, always done—3). The interpretation of the results for the total scale is (never done—0, sometimes done—1 to 44, often done—45 to 89 and always done 90 points), while for subscale fluid intake and excretion, it is (never done—0, sometimes done—1 to 14, often done—15 to 29 and always done 30 points), subscale assessment and information and subscale documentation (never done—0, sometimes done—1 to 8, often done—9 to 17 and always done 18 points), while for subscale support (never done—0, sometimes done—1 to 12, often done—13 to 23 and always done 24 points) [26]. The measure of internal consistency in previous research for practical subscales was 0.72, 0.81, 0.74 and 0.69, respectively [20,23].

A standard translation procedure, including forward, back translation, and reconciliation, was applied to ensure the semantic validity of the questionnaire [27]. All the translators and the Serbian professional proofreader conciliated with the final versions of both questionnaires. After that, a panel of experts consisting of two nurses, two nursing assistants and one physician was met to assess their face validity. They were asked to evaluate whether the items in both questionnaires were clear, unambiguous, correctly written, and at an appropriate level of difficulty for the nursing professional. Additionally, they assessed whether the instructions on the questionnaire were adequately given. The percentage of “yes” answers was 96%, suggesting that the final Serbian version of the questionnaire can be accepted [28].

Cronbach’s alpha coefficient was used to measure internal consistency and determine the reliability of the instruments. Cronbach’s alpha coefficient of the UIQ was 0.78, which indicates good reliability. The UKPI showed very good reliability; for the knowledge scale, it was 0.89, while for the practice scale, Cronbach’s alpha coefficient was calculated for each subscale; thus, for subscale fluid intake and excretion, it was 0.91; for subscale assessment and information, it was 0.89; subscale documentation 0.73 and subscale support it was 0.85.

### 2.4. Data Analysis

Data analysis was performed using descriptive and inferential statistics. The normality of the data distribution was assessed using the Kolmogorov-Smirnov test. Numerical characteristics are presented using descriptive statistics methods, such as mean values (arithmetic mean), measures of variability (standard deviation, minimum and maximum), and attributive characteristics by absolute and relative frequency. The significance of the differences was determined using the independent sample Student’s t-test and the ANOVA test with the appropriate follow-up test (post hoc test). The effect size was used as a statistical indicator that provides better insight into the study results. Cohen’s d and eta-squared (ƞ^2^) interpreted the effect size for numeric characteristics using the following interpretation: d = 0.2 small effect, d = 0.5 medium effect, and d ≥ 0.8 large effect or ƞ^2^ = 0.01 small effect, ƞ^2^ = 0.06 medium effect, and ƞ^2^ = 0.14 large effect. Pearson’s linear correlation coefficient (r) was used to determine the degree of association between students’ knowledge and attitudes about UI. Cronbach’s alpha coefficient was used to check the reliability of the UIQ and the UKPI. Statistical processing and analysis of the obtained results were performed using the software package IBM SPSS 28 Statistics, and all tests were two-sided with a significance level of *p* < 0.05.

### 2.5. Ethical Considerations

The study was approved by the management of nursing homes and obtained from the Faculty of Medicine Commission for the Ethics of Clinical Research, the University of Novi Sad, Serbia 01-39/252 of 26 August 2024. Nursing professionals’ consent to participate in the study was obtained following the Declaration of Helsinki.

## 3. Results

### 3.1. General Characteristics of Nursing Professional

Out of the total participants, n = 146 (85.4%) were female and were between 19 and 63 years old, with the average age M = 41.5 SD = 10.6. Most of them, n = 155 (90.6%), had completed secondary school, while n = 16 (9.4%) had completed high school (undergraduate education). The average total working experience of the participants was M = 15.0 SD = 10.3, and working experience in the nursing home was M = 10.1 SD = 9.2. Also, n = 145 (84.8%) stated that they did not have a continuing education course on UI, and almost as many n = 136 (79.5%) expressed an interest in learning more about UI. Other general characteristics of nurses and nurse assistants who participated in the study are shown in Table 1.

### 3.2. Nursing Professional’s Knowledge About Urinary Incontinence

The mean score on the UIQ for the whole sample was (M = 5.9 SD = 2.8) out of a maximum of 14. Nurses achieved a higher mean score (M = 6.2, SD = 2.7) than nursing assistants, but the difference was not significant (t = 1.55, *p* = 0.12).

The analysis of correct answers to the UIQ items (Table 2) showed that nurses and nursing assistants wrongly consider UI results of normal ageing (18.6% vs. 8.2%) and that most physicians ask their older patients whether they have bladder control problems (2.3% vs. 4.7%). However, they knew that women are more likely than men to develop urinary incontinence (67.4% vs. 55.3%) and that some exercises can help control urine if one leaks when one coughs, sneezes or laughs (89.5% vs. 76.5%). In addition, a low percentage of correct answers was observed on the treatment and UI effect subscale. Namely, 31.6% of the nursing professionals believed that once people start to lose control of their urine on a regular basis, they usually never regain complete control over it again, and 42.1% stated that the best treatment for involuntary urine loss is usually.

The mean score on the UKPI knowledge scale was M = 11.2 (SD = 3.5) out of a maximum of 18. A significant difference (t = 3.88, *p* = 0.00) in the nurses’ (M = 12.2, SD = 2.4) and nursing assistants’ knowledge levels (M = 10.2, SD = 4.0) was revealed, where Cohen’s d value of 0.6 indicates a medium effect size.

Most nurses and nursing assistants were familiar with the causes of UI in older patients. Namely, 88.3% of them knew that a bladder infection can cause UI and then that older men may suffer from UI after prostate surgery (87.1%). Likewise, two-thirds of the nursing professionals knew that having a stroke may lead to UI (77.8%) and that older people who have Parkinson’s are often incontinent (76.6%). In addition, 81.9% of nursing professionals recognised that UI improves in most residents with suitable treatment and that toilet training can improve incontinence in older people (78.4%) (Table 3).

### 3.3. Nursing Professional’s Practice Regarding UI

The mean score on the UKPI practice scale for the whole sample was M = 53.2 (SD = 15.9) out of a maximum of 90, which indicates that nursing home employees often implement continence care interventions (Table 4). The mean scores for the practice subscales indicate that nursing assistants, unlike nurses, only sometimes document continence care interventions M = 7.6 (SD = 4.1) and provide support for residents M = 12.0 (SD = 5.5). However, nursing assistants more often implement continence care interventions from domain fluid intake and excretion M = 20.1 (SD = 6.6) than nurses M = 16.9 (SD = 7.2), and that difference is significant (t = 3.04, *p* = 0.00). Cohen’s d value of 0.5 indicates a medium effect size.

Descriptive analysis of the UKPI practice scale answers for the whole sample revealed that 50.9% never set up a toilet plan for demented, incontinent residents (if they do not already have one). An equal number (82, 48%) never set up a toilet plan for incontinent residents who have no or only slight mental incapacity and never request that incontinent residents use the toilet according to the toilet plan if they have a toilet plan. Also, 18.7% of nursing professionals never, if the resident is demented, control how much urine they lose in an incontinence episode (damp underpants, wet pads). In comparison, 72.5% reported that they always know whether the resident wears a pad or other protective device, and 34.5% record in the documentation when the residents are incontinent (Supplemental File S1).

### 3.4. Differences in Knowledge and Practice Regarding UI to Sociodemographic Characteristics

A significant difference in nursing professionals (nurses and nursing assistants) knowledge about UI, which was assessed with the UIQ and knowledge scale of UKPI, was obtained concerning whether they had a continuing education course on UI (t = 2.25, *p* = 0.03) and whether they were interested in learning more about UI (t = 4.13, *p* = 0.00) or they are not. Namely, nurses and nursing assistants who had a continuing education course on UI and were interested in learning more about UI had higher average scores than those who did not (Table 5).

The analysis of the difference in the knowledge assessed by UIQ showed that nursing professionals differ significantly with the education level (t = 2.27, *p* = 0.03), whereby those who have completed high school achieved a better mean score M = 7.4 (SD = 3.3) than nursing professionals with secondary school education M = 5.7 (SD = 2.7). Cohen’s d value of 0.5 and 0.6 indicates a medium effect size. Also, those with longer work experience (>12 years) in the nursing home had a higher level of knowledge M = 6.6 (SD = 2.8.) with ƞ^2^ of 0.04, suggesting a small effect size.

The analysis of knowledge assessed with the knowledge scale of UKPI revealed a significant difference in the profession, whereby nurses had a higher mean score compared to nursing assistants M = 12.2 (SD = 2.4) vs. M = 10.2 (SD = 4.0). No significant differences in knowledge were observed with other sociodemographic characteristics.

Also, no significant differences were observed in the practice of UI.

### 3.5. Correlation Between Knowledge and Practice Regarding UI

The correlation between nurses’ and nursing assistants’ knowledge and practice regarding UI was explored using the Pearson linear correlation coefficient (r). A statistically significant but weak positive correlation was found between knowledge assessed by UIQ, the UKPI knowledge scale and the UKPI practice scale (Table 6). However, the correlation between knowledge and subscale assessment and information was positive and moderate, with r = 0.28 and r = 0.34.

## 4. Discussion

This study offers important insights into the knowledge and practice of nursing professionals regarding urinary incontinence (UI) in Serbian nursing homes. Namely, despite UI’s high prevalence among nursing home residents and its association with considerable physical, emotional, and financial burdens, it remains an underreported and undertreated condition in long-term care settings. One of the main causes of this could be insufficient knowledge and practice of healthcare professionals, particularly nursing professionals, which is not in line with current recommendations.

The UIQ and the UKPI knowledge scale were used to assess UI knowledge, and both instruments had good reliability in this study (α = 0.78 vs. α = 0.89). However, the UIQ is primarily designed to assess the general population’s, particularly women’s, knowledge of UI [22,23,24,25,29], while the UKPI knowledge scale is specifically designed to evaluate nursing professionals’ knowledge about UI in nursing home residents [26]. Although nurses and nursing assistants scored better on the UKPI knowledge scale than on the UIQ (62.2% vs. 42.1% of correct answers), our study findings reveal a significant knowledge gap, showing a limited comprehension and understanding of different facts and information about UI. According to the mean score on UIQ (5.9 ± 2.8), nursing professionals in Serbian nursing homes showed almost the same level of knowledge as nursing home caregivers in Turkey [18]. Unfortunately, there is still a misconception that UI is one of the results of normal ageing and that most people will involuntarily or accidentally lose control of their urine regularly by the time they are 85. Although the frequency of UI increases with ageing, the acceptance that UI is an integral part of ageing leads to the lack of adequate assessment of the nursing home residents, standard continence care and start of treatment, which may have consequences for the physical and psychosocial well-being of nursing home residents [30,31]. Another significant issue observed in this study regarding UI is that most nursing professionals suppose that physicians ask older patients if they have problems with bladder control, as well as their conviction that patients talk to their physician about urinary leakage. Such results were also obtained in the study by Yenişehir et al. [18], which indicates the need for interprofessional education with patient involvement better to understand roles and responsibilities within the medical team and indirectly improve the quality of continence care [12,32].

However, nursing professionals in Serbian nursing homes showed a higher level of knowledge about UI among older people according to the mean score of UKPI of 11.2 ± 3.5. They had particularly satisfactory knowledge regarding risk factors for UI, such as bladder infection, prostate surgery, stroke and Parkinson’s disease. However, more than 30% of nursing professionals did not know that diabetes and some antihypertensive or sleep medications can cause UI. An equal percentage were unaware that certain medications can treat urinary incontinence, while three-quarters recognised that toilet training can improve incontinence in older adults. Nurses and nursing assistants in Swiss and Turkish nursing homes demonstrated comparable levels of knowledge, as assessed using the same knowledge tool [2,18].

On the contrary, in a study conducted about two decades ago, the level of knowledge about UI of nurses in the United States of America is significantly higher than that of nurses in Serbia [33]. Perhaps one of the reasons for this might be a different education system. Namely, although nurses in Serbia can have a university education, there is still no specialist education such as Nurse Continence Specialist (NCS) [34] or advanced nurse practice program for urotherapists [21]. The study’s findings underscore the need for targeted educational programs focusing on the latest evidence-based practices in continence care or the importance of integrating certified programs on continence care into nursing education in Serbia. Namely, nursing professionals with a higher level of education and a continuous education course on UI, which is otherwise necessary for recertification, and those interested in learning more about UI had significantly better achievements on the knowledge scale.

The gap in nursing professionals’ practice was also evident in all areas, but it was the most prevalent in areas such as the documentation and support in providing continence care interventions. However, there was no significant difference in practice concerning the sociodemographic characteristics of the nursing professionals, except in the fluid intake and excretion subscale to the profession. Explicitly, nursing assistants had a significantly better mean score (*p* = 0.00) than nurses. A possible explanation for this finding could be that nursing assistants in Serbian nursing homes have longer contact with residents due to performing physical care and ADLs.

However, the fact that more than 40% of nurses and nursing assistants confirmed that they often or sometimes do not know how much the residents drink or whether the residents drink beverages with caffeine or other beverages with diuretic effects indicates that conservative UI treatment related to on fluid intake is not effectively provided. Effectively conservative treatment implies an average daily fluid intake of 1.5 L, with variations concerning illness, age, weight, activity level, and weather conditions, followed by avoiding or decreasing the intake of beverages with caffeine or other diuretic effects (coffee, cola, tea). Insufficient fluid or caffeine consumption stimulates the bladder, leading to urinary tract infection, frequency, and urgency [4,11].

In analysing the practices of nursing home professionals in Serbia, it becomes evident that they lack awareness of key information regarding residents’ incontinence. Specifically, professionals are generally unaware of the frequency of residents’ incontinence episodes, the volume of urine lost during these episodes, and how often incontinent residents go to the toilet. Consequently, essential data on excretion and fluid intake are not documented in residents’ documentation. Findings from the support subscale further indicate a lack of comprehensive toileting plans for residents with and without cognitive impairments, highlighting the absence of structured continence care interventions in our nursing home facilities. The International Continence Society recommends behavioural interventions such as prompted voiding, habit training, timed or scheduled voiding, and combined toileting and exercise therapy for managing UI in older adults, as these methods are effective and without side effects [35,36]. This finding is consistent with research that suggests that knowledge deficits can lead to less frequent or ineffective implementation of continence care interventions [37,38].

It is important to emphasise that most nursing professionals in our study knew whether the resident wore a pad or other protective devices. On the one hand, this may suggest that absorbent products are wrongly used as UI treatment options instead of only as symptom coping. On the other hand, it may indicate an organisational issue, considering that the average time needed to change absorbent products is shorter than that required to help a resident to use the toilet to urinate. Therefore, treating UI in nursing home residents is challenging [4].

One notable finding is the positive correlation between knowledge and practice, albeit weak. This suggests that while knowledge is crucial, it alone is insufficient to improve practice outcomes significantly. Other factors like workload, lack of resources, and institutional policies such as lack of authority may also play critical roles in shaping practice [39]. Consequently, interventions to improve continence care should focus on enhancing knowledge and addressing these systemic barriers [4]. The study exploring training needs regarding urinary incontinence in nursing homes revealed that nursing professionals preferred convenient teaching materials, online video training and face-to-face guidance from preceptors while combining theory and practice [19]. One approach to addressing systemic barriers in implementing UI treatment among older nursing home residents is interprofessional collaboration and partnerships between advanced nurse practitioners (urotherapists) and physicians [21,37].

### Limitations

The study has several limitations that should be considered when interpreting the findings. The cross-sectional design limits the ability to establish causal relationships between knowledge and practice. Additionally, the study was conducted in a specific region of Serbia, which may limit the generalizability of the findings to other countries or healthcare settings. Moreover, self-reported data may be biased, as participants might have overestimated their knowledge and practice due to social desirability bias.

## 5. Conclusions and Future Directions

The study’s results indicate that although nurses generally demonstrated higher knowledge levels than nurse assistants, the overall knowledge in both groups remains suboptimal. The misconception that UI is an integral part of ageing is still present. A satisfactory level of knowledge was observed in the understanding of risk factors for UI, but continence care practice, especially that related to fluid intake, documentation and toileting plans in Serbian nursing homes, is insufficient. A weak but significant positive correlation was found between knowledge and practice.

This study highlights the need for improved education and training on UI in nursing homes. Both nurses and nurse assistants must be equipped with the knowledge and skills to provide high-quality continence care. Future research should focus on evaluating the effectiveness of educational interventions and exploring the systemic factors that hinder the implementation of best practices in UI management. Additionally, longitudinal studies could provide further comprehension of the long-term effects of improved knowledge and practice on patient outcomes in nursing homes.

## Figures and Tables

**Table 1:** Nursing professional’s sociodemographic characteristics.

Variable	Whole Sample			Nurses			Nursing Assistants		
	n (%)			n (%)			n (%)		
**Sex**									
Male	25 (14.6)			18 (20.9)			7 (8.2)		
Female	146 (85.4)			68 (79.1)			78 (91.8)		
**Education level**									
Secondary school	155 (90.6)			74 (86.0)			81 (95.3)		
Undergraduate education	16 (9.4)			12 (14.0)			4 (4.7)		
**The continuing education course on UI**									
Yes	26 (15.2)			18 (20.9)			8 (9.4)		
No	145 (84.8)			68 (79.1)			77 (90.6)		
**Interest in learning more about UI**									
Yes	136 (79.5)			75 (87.2)			11 (12.8)		
No	35 (20.5)			11 (12.8)			24 (28.2)		
	**Min**	**Max**	**Mean (SD)**	**Min**	**Max**	**Mean (SD)**	**Min**	**Max**	**Mean (SD)**
**Age**	19.0	63.0	41.5 (10.6)	21.0	63.0	40.2 (11.7)	19.0	61.0	43.0 (9.4)
**Total years of working experience**	1.0	43.0	15.0 (10.3)	1.0	43.0	16.0 (11.6)	1.0	39.0	15.0 (10.3)
**Years of working experience in a nursing home**	1.0	41.0	10.1 (9.2)	1.0	41.0	11.9 (10.6)	1.0	39.0	8.3 (7.4)

**Table 2:** Distribution of correct answers on the UIQ.

Item	Total Sample n (%)	Nurses n (%)	Nursing Assistants n (%)
**Subscale 1. The relationship between ageing and UI**			
Involuntary loss of urine, often called leaky bladder or urinary incontinence, is one of the results of normal ageing.	23 (13.5)	16 (18.6)	7 (8.2)
2. Most people will involuntarily or accidentally lose control of their urine regularly by the time they are 85.	57 (33.3)	29 (33.7)	28 (32.9)
**Subscale 2. The causes of UI**			
3. Many over-the-counter medications can cause involuntary urine loss.	82 (48.0)	41 (47.7)	41(48.2)
8. Women are more likely than men to develop urinary incontinence.	105 (61.4)	58 (67.4)	47 (55.3)
10. Involuntary urine loss is caused by only one or two conditions.	84 (49.1)	51 (59.3)	33 (38.3)
12. Involuntary loss of urine can be caused by several easily treatable medical conditions.	93 (54.4)	48 (55.8)	45 (52.9)
**Subscale 3. The discussion between the patient and physician about UI**			
7. Most physicians ask their older patients whether they have bladder control problems.	6 (3.5)	2 (2.3)	4 (4.7)
9. Most people with involuntary urine loss talk to their physicians about it.	38 (22.2)	24 (27.9)	14 (16.5)
**Subscale 4. Treatment and effects of UI**			
4. Other than pads, diapers, and catheters, little can be done to treat or cure involuntary urine loss.	86 (50.3)	43 (50.0)	43 (50.6)
5. Once people start to lose control of their urine on a regular basis, they usually never regain complete control over it again.	54 (31.6)	23 (26.7)	31 (36.5)
6. Most people who currently have involuntary urine loss live normal lives.	86 (50.3)	44 (51.2)	42 (49.2)
11. Many people with involuntary urine loss can be cured, and almost everyone can experience significant improvement.	80 (46.8)	40 (46.5)	40 (47.1)
13. The best treatment for involuntary urine loss is usually surgery.	72 (42.1)	39 (45.3)	33 (38.8)
14. There are exercises that can help control urine if one leaks when one coughs, sneezes, or laughs.	142 (83.0)	77 (89.5)	65 (76.5)

**Table 3:** Distribution of correct answers on the UKPI knowledge scale.

Item	Whole Sample n (%)	Nurses n (%)	Nursing Assistants n (%)
Urinary incontinence improves in most residents with suitable treatment.	140 (81.9)	75 (87.2)	65 (76.5)
Most people need to empty the bladder every 2–4 h when awake.	136 (79.5)	73 (84.9)	63 (74.1)
Stress incontinence is caused by psychological problems.	123 (71.9)	67 (77.9)	56 (65.9)
A bladder infection can cause urinary incontinence.	151 (88.3)	81 (94.2)	70 (82.4)
Older men may suffer from urinary incontinence after prostate surgery.	149 (87.1)	79 (91.9)	70 (82.4)
Urinary incontinence can occur more often when sneezing, coughing, or walking.	127 (74.3)	68 (79.1)	59 (69.4)
On admission to a home, more women are incontinent than men.	92 (53.8)	54 (62.8)	38 (44.7)
More than 80% of all residents in nursing homes suffer from urinary incontinence.	102 (59.6)	54 (62.8)	48 (56.5)
Diabetes can cause urinary incontinence.	119 (69.6)	69 (80.2)	50 (58.8)
Having a stroke may lead to urinary incontinence.	133 (77.8)	79 (91.9)	54 (63.5)
More residents suffer from urinary incontinence after being in a nursing home for a year than at admission.	54 (31.6)	34 (39.5)	20 (23.5)
Mobility-limited residents are equally often urinary incontinent as mobile residents.	94 (55.0)	48 (55.8)	46 (54.1)
Certain medications can treat urinary incontinence.	111 (64.9)	57 (66.3)	54 (63.5)
Older people who have Parkinson’s are also often incontinent.	131 (76.6)	70 (81.4)	61 (71.8)
Some antihypertensive or sleep medications can cause urinary incontinence.	115 (67.3)	64 (74.4)	51 (60.0)
Urinary incontinence is a normal part of ageing (over 65 years).	115 (67.3)	51 (59.3)	64 (75.3)
Toilet training can improve incontinence in older people requiring care.	134 (78.4)	70 (81.4)	64 (75.3)
Demented residents are more often urinary incontinent than non-demented residents.	125 (73.1)	69 (80.2)	56 (65.9)

**Table 4:** Nursing professional’s practice regarding UI.

Subscale	Total Sample M (SD)	Nurses M (SD)	Nursing Assistants M (SD)	t _(df)_	*p*	*d*
Fluid intake and excretion (10 items/30 points)	18.5 (7.1)	16.9 (7.2)	20.1 (6.6)	3.04 _(169)_	0.00	0.5
Assessment and information (6 items/18 points)	14.4 (3.9)	14.8 (3.7)	14.1 (4.0)	1.11 _(169)_	0.27	ns
Documentation (6 items/18 points)	7.8 (4.2)	8.1 (4.3)	7.6 (4.1)	0.77 _(169)_	0.44	ns
Support (8 items/24 points)	12.5 (5.8)	12.9 (6.0)	12.0 (5.5)	1.00 _(169)_	0.32	ns
Total scale	53.2 (15.9)	52.7 (17.4)	53.9 (14.5)	0.48	0.63	ns

**Table 5:** Differences in knowledge and practice of nursing professionals regarding UI to sociodemographic characteristics.

	UIQ				UKPI (Knowledge Scale)				UKPI (Practice Scale)			
	M ± SD	t/F _(df)_	*p*	d/ƞ^2^	M ± SD	t/F _(df)_	*p*	d/ƞ^2^	M ± SD	t/F _(df)_	*p*	d/ƞ^2^
**Gender**												
Male	6.5 ± 3.0	1.22 _(169)_	0.22	ns	11.8 ± 3.7	0.96 _(169)_	0.34	ns	53.3 ± 14.9	0.13 _(169)_	0.90	ns
Female	5.8 ± 2.7				11.1 ± 3.4				52.9 ± 21.6			
**Educational level**												
Secondary school	5.7 ± 2.7	2.27 _(169)_	**0.03**	0.6	11.1 ± 3.5	1.00 _(169)_	0.27	ns	52.6 ± 15.7	1.83 _(169)_	0.07	ns
High school	7.4 ± 3.3				12.1 ± 2.4				60.2 ± 16.9			
**Profession**												
Nurses	6.2 ± 2.7	1.55 _(169)_	0.12	ns	12.2 ± 2.4	3.88 _(169)_	**0.00**	0.6	52.7 ± 17.4	0.48 _(169)_	0.63	ns
Nursing assistants	5.6 ± 2.9				10.2 ± 4.0				53.9 ± 14.5			
**Working experience in a nursing home**												
<4 years	5.9 ± 2.7	3.50 _(2)_	**0.03**	0.04	10.9 ± 3.6	2.80 _(2)_	0.64	ns	54.2 ± 16.6	0.82 _(2)_	0.82	ns
4.1–12 years	5.2 ± 2.7				10.7 ± 4.0				53.1 ± 14.6			
>12 years	6.6 ± 2.8				12.1 ± 2.6				52.3 ± 16.9			
**The continuing education course on UI**												
Yes	7.0 ± 2.7	2.15 _(169)_	**0.03**	0.5	12.6 ± 2.6	2.25 _(169)_	**0.03**	0.5	56.0 ± 17.3	0.96 _(169)_	0.34	ns
No	5.7 ± 2.8				10.1 ± 3.5				52.8 ± 15.7			
**Interest in learning more about UI**												
Yes	6.2 ± 2.7	3.03 _(169)_	**0.00**	0.6	11.8 ± 2.7	4.13 _(169)_	**0.00**	0.6	54.2 ± 15.5	1.53 _(169)_	0.13	ns
No	4.7 ± 2.9				9.2 ± 5.0				49.6 ± 17.5			

M = mean; SD = standard deviation; t = t-test; df = degrees of freedom; *p*-value; bold = significant; ns = not significant; d = Cohen’s d indicator, ƞ^2^ = partial eta squared.

**Table 6:** Correlation of mean score on UIQ, Knowledge scale of UKPI, Practice scale of UKPI and subscales.

	UIQ	Knowledge Scale of UKPI
Pearson (r)		
Practice scale of KPI	0.195 *	0.151 *
Fluid intake and excretion	0.141	0.094
Assessment and information	0.277 **	0.344 **
Documentation	0.076	0.153 *
Support	0.123	0.191 *

* Correlation is significant at the 0.05 level (2-tailed); ** Correlation is significant at the 0.01 level (2-tailed).

## Data Availability

The data supporting this study’s findings are available on request from the corresponding author.

## Supplementary Materials

The following supporting information can be downloaded at: https://www.mdpi.com/article/10.3390/healthcare12232425/s1, Supplemental File S1: Distribution of answers on the Practice scale of UKPI for the whole sample.
