# Supplementary material for: Knowledge and Practice of Urinary Incontinence Management Among Nursing Professionals in Serbian Nursing Homes: A Multicentre Study
Source: Healthcare (Basel). 2024 Dec 3;12(23):2425. doi: 10.3390/healthcare12232425 (PMC11641492; doi:10.3390/healthcare12232425)
Supplement: Supplementary file 1 [file healthcare-12-02425-s001.zip › healthcare-3329049-supplementary.pdf]

Supplemental file S1. Distribution of answers on the Practice scale of KPI for the whole sample

Item	Never<br>n (%)	Sometimes<br>n (%)	Often<br>n (%)	Always<br>n (%)
**Fluid intake and excretion**				
I know how much the residents drink	14 (8.2)	53 (31.0)	59 (34.5)	45 (26.3)
I know the times at which the residents drink.	9 (5.3)	62 (36.3)	71 (41.5)	29 (17.0)
I know whether the residents drink beverages with caffeine or other beverages with diuretic effects (e.g., coffee, cola etc.).	10 (5.8)	46 (26.9)	65 (38.0)	50 (29.2)
I know how much urine the residents lose in an incontinence episode (damp underpants, wet pads, etc.).	24 (14.0)	42 (24.6)	61 (35.7)	44 (25.7)
If the resident is demented, I control how much urine they lose in an incontinence episode (damp underpants, wet pads, etc.).	32 (18.7)	48 (28.1)	41 (24.0)	50 (29.2)
I know how often the incontinent residents go to the toilet (e.g., every 30 min, every 3 hours, etc.).	18 (10.5)	69 (40.4)	52 (30.4)	32 (18.7)
I know the situations (e.g., during morning coffee, taking a walk, bathing, etc.) where the residents are incontinent/lose urine.	16 (9.4)	60 (35.1)	47 (27.5)	48 (28.1)
I know the frequency of incontinence episodes of the residents (e.g., once a week, several times a day, etc.).	16 (9.4)	58 (33.9)	61 (35.7)	36 (21.1)
If the resident has dementia, I check the times at which he/she loses urine.	17 (9.9)	24 (14.0)	39 (22.8)	91 (53.2)
I know whether the resident wears a pad or other protective devices.	6 (3.5)	17 (9.9)	24 (14.0)	124 (72.5)
**Assessment and information**				
On admission and if the health status changes, I ask the resident how long he/she has been incontinent.	1 (0.6)	10 (5.8)	51 (29.8)	109 (63.7)
I ask the residents whether they notice that they lose urine.	3 (1.8)	22 (12.9)	57 (33.4)	89 (52.0)
I ask incontinent residents about secondary episodes in using the toilet (such as pain on urinating, feeling of urgency, dripping).	5 (2.9)	28 (16.4)	45 (26.3)	93 (54.4)
I ask residents whether they lose urine especially on sneezing, laughing or coughing.	8 (4.7)	34 (19.9)	43 (25.1)	86 (50.3)
If a resident become incontinent, I inform the physician.	7 (4.1)	11 (6.4)	19 (11.1)	134 (78.4)
If the resident was already incontinent at home, I ask what she/he did about it and whether it helped.	6 (3.5)	28 (16.4)	40 (23.4)	97 (56.7)

Item	Never n (%)	Sometimes n (%)	Often n (%)	Always n (%)
**Documentation**				
I note in the documentation the times at which the residents are incontinent.	28 (16.4)	59 (34.5)	24 (14.0)	60 (35.1)
I note in the documentation how much urine the resident loses in an incontinence episode.	43 (25.1)	63 (36.8)	29 (17.0)	36 (21.1)
It makes no sense to write down how much the incontinent resident's drink.	64 (37.4)	58 (33.9)	35 (20.5)	14 (8.2)
I note in the documentation the times at which the incontinent residents drink.	48 (28.1)	70 (40.9)	21 (12.3)	32 (18.7)
I note in the documentation what the incontinent residents drink.	47 (27.5)	69 (40.4)	21 (12.3)	34 (19.9)
If demented incontinent residents express a wish to go to the toilet, I tell them they can go in the pads.	51 (29.8)	50 (29.2)	27 (15.8)	43 (25.1)
**Support**				
I inform the ward if a resident becomes incontinent.	6 (3.5)	14 (8.2)	15 (8.8)	136 (79.5)
I set up a toilet plan for incontinent residents who have no or only slight mental incapacity.	82 (48.0)	41 (24.0)	27 (15.8)	21 (12.3)
I set up a toilet plan for demented, incontinent residents (if they do not already have one).	87 (50.9)	40 (23.4)	24 (14.0)	20 (11.7)
If incontinent residents have a toilet plan, I request that they use it according to the plan.	82 (48.0)	36 (21.1)	30 (17.5)	23 (13.5)
If demented, incontinent residents have a toilet plan, I request that they use the toilet according to the toilet plan.	76 (44.4)	38 (22.2)	31 (18.1)	26 (15.2)
I help mobility-limited incontinent residents to use the toilet according to the toilet plan.	38 (22.2)	34 (19.9)	47 (27.5)	52 (30.4)
If incontinent residents express a wish to go to the toilet, I help them if necessary.	16 (9.4)	23 (13.5)	42 (24.6)	90 (52.6)
If mobility-limited incontinent residents express the wish to use the toilet, I help them.	14 (8.2)	29 (17.0)	39 (22.8)	89 (52.0)
